# Neonatal growth velocity of preterm infants: The weight Z-score change versus Patel exponential model

**DOI:** 10.1371/journal.pone.0218746

**Published:** 2019-06-28

**Authors:** Laure Simon, Matthieu Hanf, Anne Frondas-Chauty, Dominique Darmaun, Valérie Rouger, Géraldine Gascoin, Cyril Flamant, Simon Nusinovici, Jean-Christophe Rozé

**Affiliations:** 1 Department of Neonatal Medicine, Nantes University Hospital, Nantes, France; 2 Epidemiologie Clinique, Centre d’Investigation Clinique (CIC004), Nantes University Hospital, Nantes, France; 3 INSERM CIC 1413, Clinical Investigation Center, Nantes University Hospital, Nantes, France; 4 INRA, UMR 1280 Physiologie des Adaptations Nutritionnelles, Nantes University Hospital, Nantes, France; 5 Réseau "Grandir Ensemble", Nantes University Hospital, Nantes, France; 6 Department of Neonatal Medicine, Angers University Hospital, Angers, France; Centre Hospitalier Universitaire Vaudois, FRANCE

## Abstract

**Background:**

Different methods are used to assess the growth of preterm infants during neonatal hospital stay. The primary objective was to compare two methods for assessing growth velocity: g/kg/d according to the Patel exponential model (EM) and change in weight z-score (ZS) according to Fenton curves. The secondary objective was to highlight factors influencing the level of agreement between the two methods.

**Methods:**

Preterm infants born before 33 weeks were included. Growth velocity was computed by EM and ZS methods and linear regression was used to predict what growth velocity by EM method would be obtained using the ZS method. Differences between EM growth velocity and EM growth velocity predicted by ZS method were then used to assess the level of agreement between the two methods. A difference between -2 and +2 g/kg/day was considered as fair agreement, greater than ± 4 g/kg/day as poor agreement, and as disagreement otherwise.

**Results:**

Among the 3954 children included, we observe a fair agreement in 2471 children (62.5%), a poor agreement in 1278 (32.3%) and a disagreement in 205 children (5.2%). Birth weight and gestational age explained 31% and 25%, respectively, of the variance in the difference between the two methods.

**Conclusions:**

In more than a third of enrolled children, the two methods for measuring growth velocity disagreed substantially. As variation of weight Z-score takes into account infant gestational age and gender, it could be more suitable to analyze a population of preterm infants with a wide range of gestational age.

## Introduction

Ensuring adequate growth in very preterm infants during neonatal hospital stay represents a challenge and a top priority for neonatologists. The aim of the nutritional management of preterm infants is to support a growth trajectory which mimics the fetal growth during the 3^rd^ trimester of gestation, which is normally associated with a tripling in fetal weight [1–2]. Growth rate in the perinatal period is associated with neurologic and metabolic outcomes [3–4]. Ehrenkranz et al. showed that a higher weight gain based on g/kg/d measurements during neonatal hospital stay was associated with better neurologic outcomes between 18 and 22 months of age in preterm infants [5]. Frondas et al. drew similar conclusions with a growth analysis based on change in weight Z-score [6].

The measurement of postnatal growth is thus central for the clinical care and investigation of very preterm infants. However, no clear consensus currently exists concerning the methods suitable to quantify growth in this population leading clinicians and researchers to use a variety of methods [7–10]. A recent systematic review concludes that more research is clearly needed in the field to identify which methods are preferable to quantify the growth of very preterm infants [8].

Two of the most frequently used methods to calculate weight gain velocity use g/kg/d and change in Z-score relative to an intrauterine or postnatal reference growth chart [8]. Over the last decade, growth velocity was frequently assessed by change of weight Z-score in spite of the fact that Patel showed that g/kg/d estimates based on an exponential model were accurate [7,11]. The latter model was used to assess the growth of preterm infants during neonatal hospitalization by several authors [10,12]. Similarly, Fenton et al. recently examined how well growth velocity recommendations for preterm infants fit with current growth references but did not include Z-score methods in their analysis [13]. Although it is necessary to evaluate whether the methodological differences between measurement methods may significantly impact the calculated growth velocity, no clear evaluation of the agreement between g/kg/d and change in Z-score was performed until now.

The aim of this study was thus to determine in preterm infants of less than 33 weeks of gestational age whether the two methods for measuring the growth velocity: g/kg/d according to the Patel exponential model (EM), and change in weight Z-score (ZS) according to Fenton curves, resulted in concordant results. The secondary objective was to highlight factors influencing the level of agreement between these two methods.

## Materials and methods

### Patients

The study population was composed of preterm infants enrolled in the Loire Infant Follow-up Team (LIFT) cohort, born at <33 weeks of gestation between January 2003 and December 2015. The LIFT network encompasses 24 maternity clinics including 5 neonatal intermediate or intensive care units in the Pays-de-la-Loire region [14]. The children’s parents provided written informed consent before inclusion in the LIFT cohort. The patient database was registered with the French data protection authority for clinical research (Commission Nationale de l’Informatique et des Libertés (CNIL)). The study was approved by the Nantes Ethics Committee (Groupe Nantais d’Ethique dans le Domaine de la Santé (GNEDS)). Verbal consent was obtained from parents, and a statement of “non opposition” was recorded in the infant’s clinical chart, as required by French law for this kind of observational study.

### Evaluation of growth and agreement between the two methods

Data on growth were recorded at birth and at discharge. Body weight was measured on an electronic scale accurate to the nearest 1g.

Growth velocity was computed by two methods, EM and ZS methods:

[1] To compute growth velocity (g/kg/d) using EM method, we calculated the exponential relationship between initial weight (W1) and weight at the second time point (Wn) as a function of time, with D representing day of life [7]. An exponential model assumes that growth occurs at a constant fraction (k) of the previous weight, such that weight changes over time by some fraction of the previous weight. To compute growth velocity by EM method, the following formula was used:

Growth velocity (g/kg/d) by EM method = [1000 x ln (discharge Weight / birth Weight)] / length of hospital stay, where ln is the natural logarithm, and weights are expressed in grams, and length of hospital stay in days.

Growth velocity by EM method was called EM growth velocity.

[2] To compute growth velocity using ZS method, we calculated Z-score by using λ-μ-σ method (LMS). We used Fenton growth chart for birth and discharge [15]. The following formula was used:

Growth velocity by ZS method = weight ZS at discharge–weight ZS at birth.

Growth velocity calculated by ZS method was called ZS growth velocity.

Because EM and ZS growth velocities are expressed in different units, linear regression was used to predict what the EM growth velocity would be, given the ZS method. Differences between EM growth velocity and EM growth velocity predicted by ZS method were then used to assess the level of agreement between the two methods.

### Statistical analysis

To compare differences between EM growth velocity and EM growth velocity predicted by ZS method, the 95% limits of agreement (1.96 standard deviation of the difference) as formalized by Bland and Altman [16] were computed. Normality of the differences was checked. Values obtained by the two methods were plotted against each other with the calculated 95% limits of agreement. Three limits of agreements were preliminary defined between the two methods based on clinical pertinence and literature. Those limits are arbitrary. A limit of agreement between ± 2 g/kg/day was considered as fair agreement, between ± 4 g/kg/day as poor agreement and as disagreement otherwise.

The difference between the two methods was visually inspected according to gender, gestational age, and birth weight ZS. Multivariable regression analyses were then used to determine the adjusted relationships between the differences and 1) characteristics of the child (gender, gestational age, birth weight, birth weight Z-score, discharge weight, discharge weight Z-score, growth velocity, weight Z-score change and parent’s socioeconomic level) 2) characteristics of the mother and her pregnancy (multiple pregnancy, antenatal corticotherapy, hypertension during pregnancy) and 3) characteristics of the neonatal hospital stay (Apgar score at 5 min, bronchopulmonary dysplasia, late onset infection and breastfeeding at discharge) as independent variables.

All the analyses were performed with the statistical software R. Significance level was set to p < 0.05. To describe the study population, medians and interquartile ranges (IQR) were computed for continuous variables and compared between groups using a Mann-Whitney test as well as proportions for categorical variables and chi-square tests for their comparisons.

## Results

### Baseline characteristics

We included 4,652 children born < 33 weeks of gestational age between January 2003 and December 2015, who had been enrolled in the LIFT network. The study population consisted of the 3,954 children (85.0%) with no missing data concerning birth weight and weight at discharge.

The baseline characteristics of the patients are described in **Table 1**. In the 3,954 included preterm children, 2099 (53.1%) were male, 278 (7.0%) had a gestational age below 26 weeks of gestation and 1950 (49.3%) above 30 weeks of gestation. Median birth and discharge weights were 1320 g (IQR: 1030; 1590 g) and 2500 g (IQR: 2200; 2800 g), respectively, with an associated median EM growth velocity of 11.4 g/kg/day (IQR: 9.9; 12.9 g/kg/day) and a median ZS growth velocity of -1.10 (-1.50; -0.60) during hospital stay.

**Table 1:** Characteristics of the population.

Variable	Category	n [%]
		N = 3,954
Children’s characteristics		
**Child’s gender, n (%)**	Male	2099 [53.1]
**Gestational age, n (%)**	23–26 wks	278 [7.1]
	27–28 wks	685 [17.3]
	29–30 wks	1041 [26.3]
	31–32 wks	1950 [49.3]
**Birth weight**	< 1000 g	939 [23.7]
	1000–1500 g	1724 [43.6]
	> 1500 g	1291 [32.7]
**Birth weight, g**	Median [IQR]	1320 [1030,1595]
**Birth weight Z-score**	Median [IQR]	-0.4 [-1.1,0.1]
**Discharge weight, g**	Median [IQR]	2520 [2200,2810]
**Discharge weight Z-score**	Median [IQR]	-1.2 [-1.8,-0.6]
**Growth velocity, g/kg/day**	Median [IQR]	11.4 [9.9,12.9]
**Weight Z-score change**	Median [IQR]	-0.7 [-1.2,-0.3]

### Agreement between EM and ZS growth velocity methods

**Fig 1:**
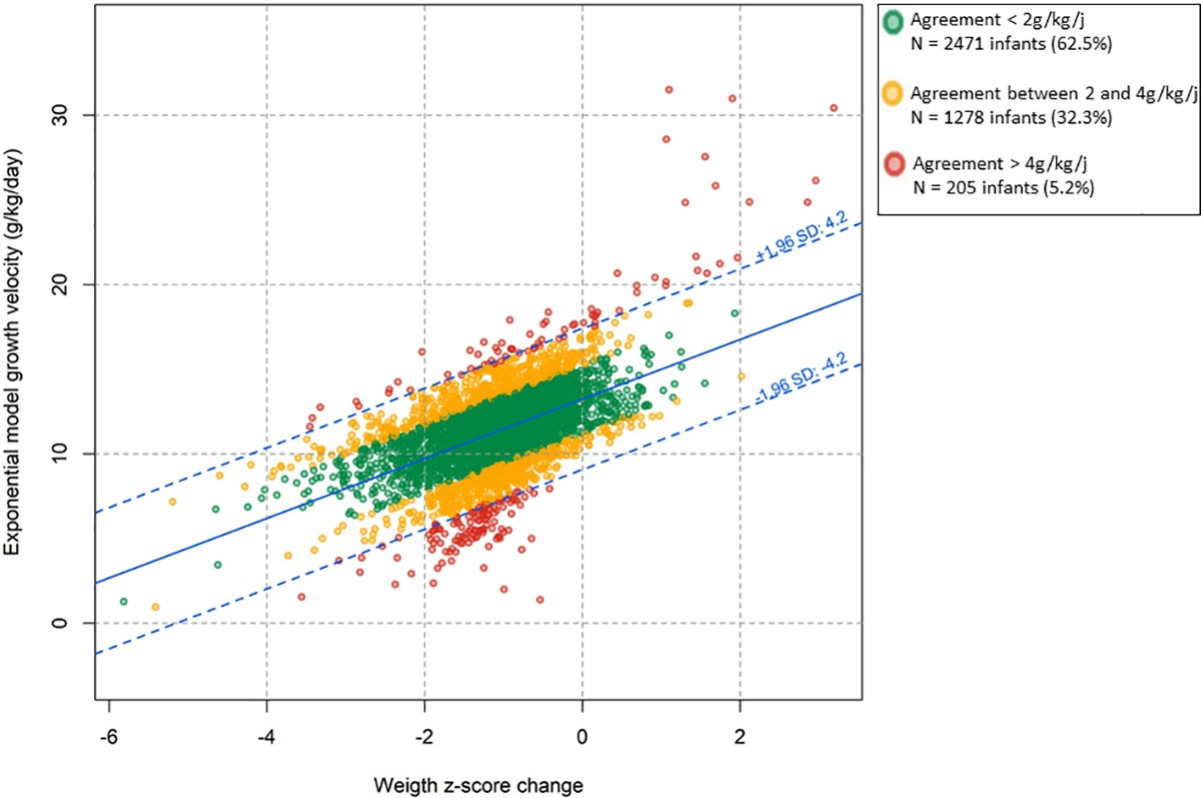
Scatterplot and 95% limits of agreement of the relationship between exponential model and Z-score growth velocities. Z-score growth velocity is the exponential model growth velocity predicted by weight Z-score change according to Fenton curves during neonatal hospital stay (n = 3,954) Green points represent infants with agreement < 2 g/kg/day, yellow points represent agreement between 2 and 4 g/kg/day and red points agreement > 4g/kg/d.

**Fig 1** shows the observed relationship between EM and ZS growth velocity values as well as the 95% limits of agreement computed according to Bland and Altman. The mean difference between EM growth velocity and EM growth velocity predicted by ZS method was 0.0 g/kg/day with a standard deviation of 2.1 g/kg/day. These differences were observed in both directions and followed a Gaussian distribution. The calculated 95% limits of agreement was ± 4.2 g/kg/day meaning that the difference between EM growth velocity and EM growth velocity predicted by ZS method was in 95% of cases within ± 4.2 g/kg/day of the observed EM growth velocity. According to our predefined cut-offs, this observed agreement between the two methods was classified as disagreement.

In the study population, 62.5% (n = 2471) of children had a difference of less than 2 g/kg/day, 32.3% (n = 1278) a difference of more than ± 2 g/kg/day, and 5.2% (n = 205) a difference of more than ± 4 g/kg/day between the two methods for measuring growth velocity.

### Identification of factors influencing the difference between EM growth velocity and EM growth velocity predicted by ZS method

**Fig 2:**
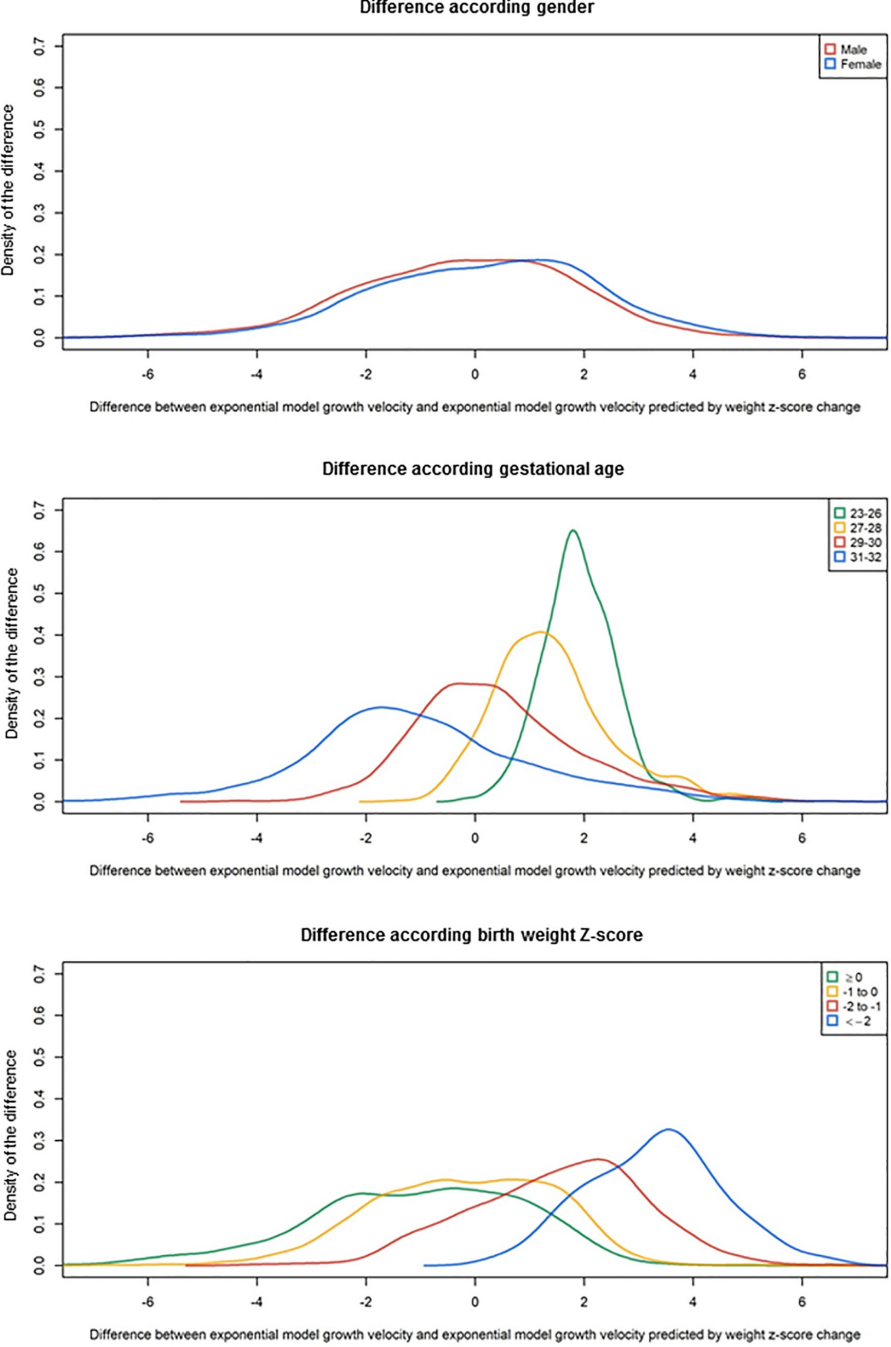
Agreement between exponential model and Z-score growth velocities according to gender, gestational age and birth weight Z-score. Z-score growth velocity is the exponential model growth velocity predicted by weight Z-score change according to Fenton curves during neonatal hospital stay according to gender, gestational age and birth weight Z-score (n = 3,954).

**Fig 2** shows the density of the differences between EM growth velocity and EM growth velocity predicted by ZS method during neonatal hospitalization according to gender, gestational age and birth weight Z-score. Results of the multiple regression analysis are shown in **Table 2**. On multivariable analysis, child’s gender, gestational age, birth weight had a significant impact on the difference between EM growth velocity and EM growth velocity predicted by ZS method (p< 0.05). In this multivariable analysis, female gender, low gestational age, low birth weight Z-score increased the observed difference between the two methods. Birth weight and gestational age explained 31% and 25%, respectively, of the variance in the difference between EM growth velocity and EM growth velocity predicted by ZS method. All others included variables explained less than 1% of the variance.

**Table 2:** Multiple regression analysis for factors associated with the difference between exponential model growth velocity and exponential model growth velocity predicted by weight Z-score change according to Fenton curves.

Variable	Category	Estimate [95%-CI]	P-value
*Children’s characteristics*			
**Child’s gender**	*Male*	0	-
	*Female*	0.22 [0.14, 0.3]	< 0.001
**Gestational age**	*23–26 wk*	3.53 [3.34, 3.72]	< 0.001
	*27–28 wk*	2.76 [2.63, 2.89]	< 0.001
	*29–30 wk*	1.72 [1.62, 1.83]	< 0.001
	*31–32 wk*	0	-
**Birth weight Z-score**	< -2	5.09 [4.8, 5.38]	< 0.001
	[-2; -1[	3.02 [2.9, 3.15]	< 0.001
	[-1; 0[	1.1 [1, 1.2]	< 0.001
	≥ 0	0	-

n = 3,954

## Discussion

In this study, we highlight the very poor agreement between two methods commonly used for neonatal growth assessment, growth velocity by EM and ZS methods. In our population, a fair agreement between the two methods was found in only 62.5% of preterm children, 32.3% had a poor agreement and 5.2% a disagreement. The cut-offs of 2 and 4 g/kg/d are arbitrary but they seem to be relevant for clinical practice. Moreover, it corresponds to the change of weight gain quartiles in the study by Ehrenkranz et al [5] (between 2.2 and 3.6 g/kg/d).

Birth weight and gestational age explained 31% and 25%, respectively, of the variance in the difference between EM growth velocity and EM growth velocity predicted by ZS method. All others included variables explained less than 1% of the variance. Difference between the two methods increased when gestational age decreased. Growth velocity during the third trimester of gestation is not linear and highly depends on weeks of gestation). The change of weight Z-score takes into account gestational age, and therefore seems more physiologically relevant to assess growth during the third trimester of gestation.

Gender is associated with the difference between EM growth velocity and EM growth velocity predicted by ZS method. Nutrition and growth may affect boys more dramatically than girls [6,17]. Boys may be more vulnerable to the stress associated with birth [18]. Studies on placentae of mothers who delivered prematurely described sex-specific alterations of pro-oxidant/antioxidant balance with a predominantly pro-oxidant status in placentae of male infants [19]. Growth trajectories therefore are different for boys and girls. Contrary to EM, ZS growth velocity according to Fenton curves takes into account the known difference between boys and girls.

Fenton standards were used to compute ZS. Other standards exist and could question the validity of our results. There is no consensus on which growth curve should be used. We have chosen to use Fenton curves for several reasons 1) the curves had been established from a large sample of newborns 2) the LMS data used to calculate weight, length and head circumference ZS in her reference curve were kindly provided by Dr Fenton; and 3] the curves are specific for boys and girls. Moreover, similar trends were however observed when using Olsen curves [20] (**S1 and S2 Figs, Table 3**) confirming the robustness of the disagreement between EM and ZS methods.

**Table 3:** Multiple regression analysis for factors associated with the difference between exponential model growth velocity and exponential model growth velocity predicted by weight Z-score change according to Olsen curves.

Variable	Category	Estimate (95%-CI)	P-value
*Children’s characteristics*			
**Child’s gender**	*Male*	0	-
	*Female*	-0.12 (-0.17, -0.08)	< 0.001
**Gestational age**	*23–26 wk*	-0.68 (-0.78, -0.58)	< 0.001
	*27–28 wk*	-0.4 (-0.47, -0.33)	< 0.001
	*29–30 wk*	-0.19 (-0.24, -0.13)	< 0.001
	*31–32 wk*	0	-
**Birth weight Z-score**	< -2	0.83 (0.74, 0.93)	< 0.001
	[-2; -1]	0.71 (0.65, 0.77)	< 0.001
	[-1; 0]	0.47 (0.41, 0.52)	< 0.001
	≥ 0	0	-

With a fair agreement between the two methods for 62.5% of preterm children, we understand that clinicians use either of the methods for clinical practice and research [8]. Nevertheless, poor agreement or disagreement was observed in 37.5% of children in our cohort. We believe the use of an exponential calculation of weight gain velocity is questionable. The rapid early growth observed in preterm infants indeed does not sustainably follow an exponential trajectory, but rather decreases rapidly after early infancy [21].We fully agree with Fenton et al who recently suggested [13] that ZS growth velocity calculation warrants consideration.

The choice of an appropriate method of growth assessment is important both for clinical practice and research. Postnatal growth is used to guide day-to-day decisions, such as determining the feeding regimen of preterm infants [22]. The lack of standardization of methods of growth assessment makes comparisons between studies difficult and represents an obstacle for the translation of results from research studies into improved clinical guidelines. It therefore appears urgent to standardize the methods for measuring growth velocity in preterm infants.

Our study suffered several limitations. First, when comparing children included and not included in the analysis, significant differences could be seen (**Table 4**). Preterm children with an antenatal corticosteroid treatment, bronchopulmonary dysplasia, and breastfeeding at discharge were indeed overrepresented in those included in the analysis. However, because 1) our study was based on a large number of children (n = 3,954) with a good distribution in all studied variables 2) the number of not included children was relatively small (n = 698, 15%), 3) no differences in anthropometric data were observed between included and not included children and 4) this restriction did not result in obvious selection bias (identified factors were indeed both positively and negatively correlated to observed differences), this bias was of limited impact.

**Table 4:** Comparison of the included and not included population.

	Not included preterm infants	Included preterm infants	Total	P value
Total	n = 698	n = 3954	n = 4652	
**Gender**				0.78
Boys	366 (52.4)	2099 (53.1)	2465 (53)	
Girls	332 (47.6)	1855 (46.9)	2187 (47)	
**Gestational age**				0.11
23–26 wks	52 (7.4)	278 (7)	330 (7.1)	
27–28 wks	117 (16.8)	685 (17.3)	802 (17.2)	
29–30 wks	213 (30.5)	1041 (26.3)	1254 (27)	
31–32 wks	316 (45.3)	1950 (49.3)	2266 (48.7)	
**Birth weight**				0.67
>1500 g	216 (30.9)	1291 (32.7)	1507 (32.4)	
<1000 g	169 (24.2)	939 (23.7)	1108 (23.8)	
1000–1500 g	313 (44.8)	1724 (43.6)	2037 (43.8)	

Another limitation concerns the number of weight measurements. However, this limitation should not bias the comparison between the methods as the same number of measurement is used for both methods. In addition to this, the postmenstrual age of discharge is not the same for all preterm infants. Nevertheless both methods take account for the length between birth and discharge, by weighting according to the length of stay in the exponential model and by calculating Z-scores at each ages. The main strength was the large sample of preterm infants enrolled and the population-based birth cohort.

## Conclusions

A fair agreement between the two methods for assessing growth velocity: EM growth velocity and EM growth velocity predicted by ZS method was observed in only 62.5% of preterm children in our cohort. Birth weight and gestational age explained the bulk of the variance in the difference between the two methods. As variation of weight Z-score takes into account both gestational age and gender, this approach could be more suitable to analyze a population with a wide range of gestational age. More studies are needed to confirm this result in other populations.

## Supporting information

S1 Fig Scatterplot and 95% limits of agreement of the relationship between exponential model and Z-score growth velocity. Z-score growth velocity is the exponential growth velocity predict by weight Z-score change according to Olsen curves during neonatal hospital stay (n = 3,954) Green points represent infants with agreement < 2 g/kg/day, yellow points agreement between 2 and 4 g/kg/day and red points agreement > 4g/kg/d.
(JPG)

S2 Fig Density of the differences between exponential model and Z-score growth velocities. Z-score growth velocity is the exponential model growth velocity predicted by weight Z-score change according to Olsen curves during neonatal hospital stay according to gender, gestational age and birth weight Z-score (n = 3,954).
(JPG)

S1 Table Raw data of the study population. (XLS)
