# Supplementary figures and images for: Neonatal growth velocity of preterm infants: The weight Z-score change versus Patel exponential model
Source: PLoS One. 2019 Jun 28;14(6):e0218746. doi: 10.1371/journal.pone.0218746 (PMC6599123; doi:10.1371/journal.pone.0218746)

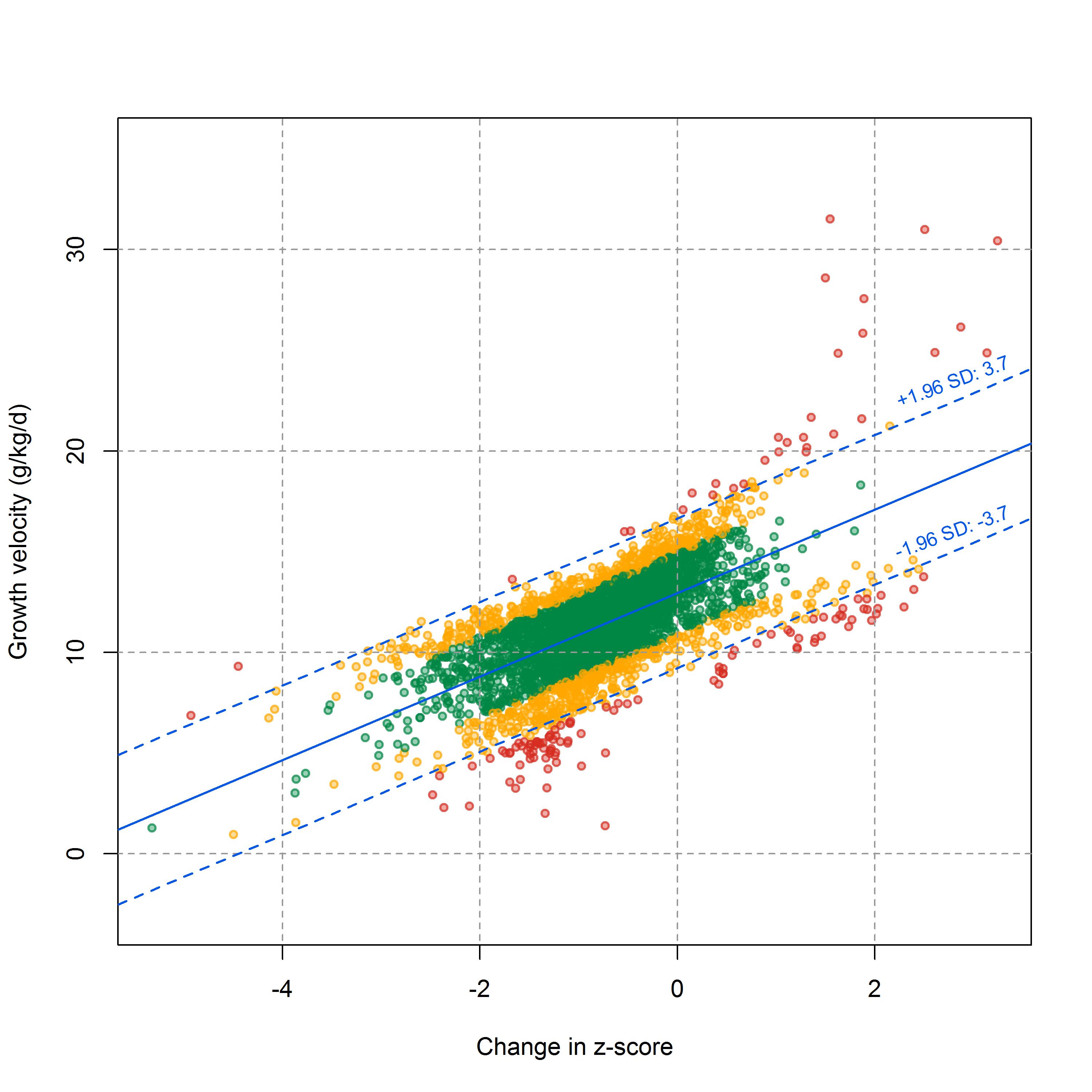

Supplement: S1 Fig — Z-score growth velocity is the exponential growth velocity predict by weight Z-score change according to Olsen curves during neonatal hospital stay (n = 3,954) Green points represent infants with agreement < 2 g/kg/day, yellow points agreement between 2 and 4 g/kg/day and red points agreement > 4g/kg/d. (JPG) [file pone.0218746.s001.jpg]

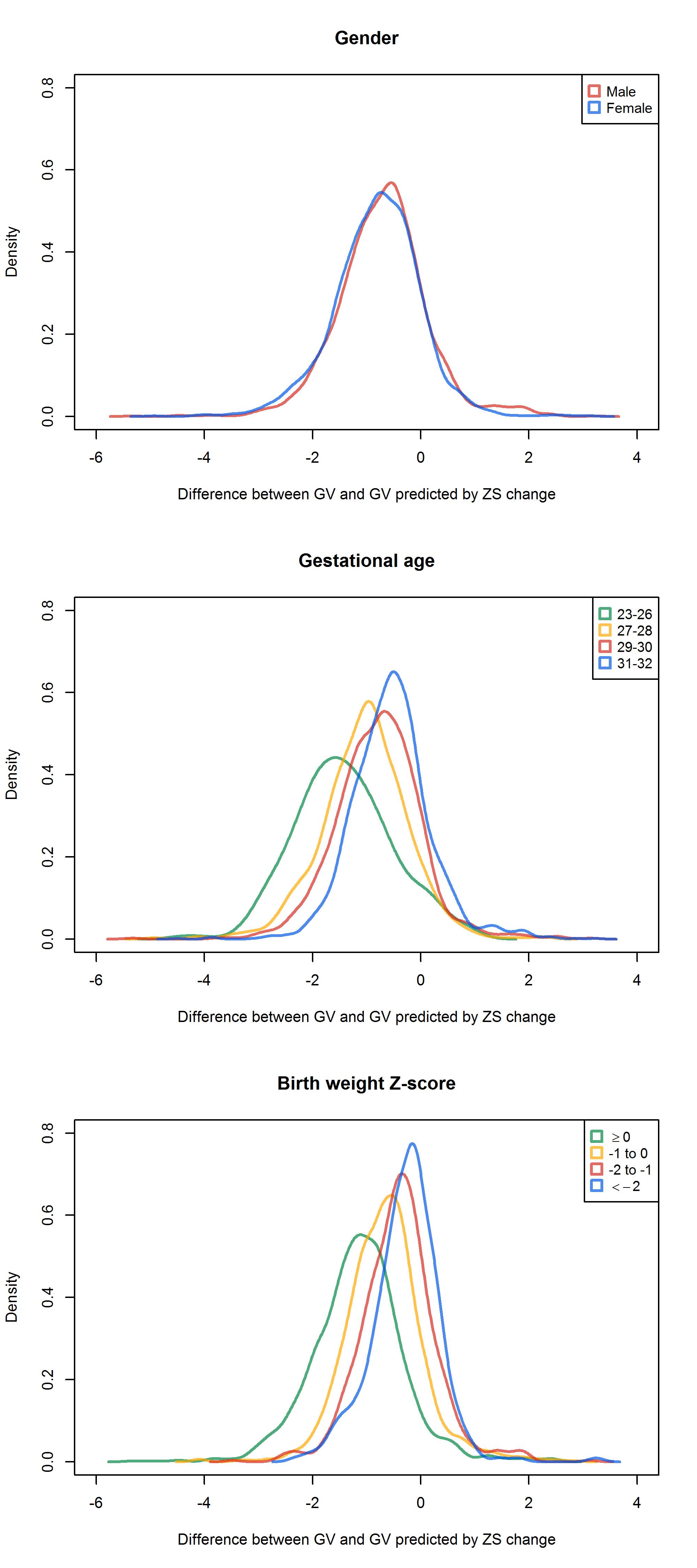

Supplement: S2 Fig — Z-score growth velocity is the exponential model growth velocity predicted by weight Z-score change according to Olsen curves during neonatal hospital stay according to gender, gestational age and birth weight Z-score (n = 3,954). (JPG) [file pone.0218746.s002.jpg]
